# Minimally invasive mastery: transorbital endoscopic excision of the paramedian epidermoid

**DOI:** 10.3171/2025.1.FOCVID24188

**Published:** 2025-04-01

**Authors:** Ravi Sankar Manogaran, Abhishek Shukla, Kalyana Sundaram Chithambaram, Anant Mehrotra, Nidhin Das K, Sushila Jaiswal, Awadhesh Kumar Jaiswal

**Affiliations:** 1 Neurotology Unit, Department of Neurosurgery, and; Departments of 2 Neurosurgery, Sanjay Gandhi Postgraduate Institute of Medical Sciences, Lucknow, India and; 3 Pathology, Sanjay Gandhi Postgraduate Institute of Medical Sciences, Lucknow, India

**Keywords:** epidermoid, cavernous sinus, TONES, orbitotomy, minimally invasive surgery

## Abstract

Endoscopic minimally invasive skull base surgery using the transorbital neuroendoscopic technique for cavernous sinus epidermoid serves as a pivotal link to open transcranial surgeries. This method involves a minimally invasive transorbital approach, including lateral orbitotomy and drilling the greater sphenoid wing, followed by cutting the orbitomeningeal band, peeling the dura mater, and exposing the tumor. This approach is technically demanding and requires thorough knowledge of anatomy and familiarity with endoscopic instruments. The favorable clinical, cosmetic, and radiological outcomes underscore the effectiveness of this technique, highlighting the role of endoscopy in minimally invasive skull base paramedian pathologies.

The video can be found here: https://stream.cadmore.media/r10.3171/2025.1.FOCVID24188

## Transcript

### 0:21 Introduction and Preparation.

This video describes a minimally invasive mastery transorbital endoscopic excision of the paramedian epidermoid. Presenting a case of a 33-year-old female with no comorbidity. Presented with a right hemicranial headache for 10 months and hemifacial hypoesthesia lasting the same duration. On examination, she had 30%–40% right hemifacial hypoesthesia in V1, V2, and V3 distribution, as well as right masseter muscle atrophy and temporal muscle hollowing on motor examination. On radiological examination, there was a well-defined right parasellar expansile lesion with an eroded petrous apex and extension into the posterior fossa on the bony window. A single hypodense, non–contrast-enhancing lesion was noted along the lateral cavernous sinus with posterior fossa extension on soft tissue window. T1 contrast axial section MRI showed a single nonenhancing, well-defined hypointense lesion occupying the right cavernous area with posterior fossa extension. The same lesion on T2-weighted MRI appears hyperintense, without any flow voids within the lesion, and shows diffusion restriction on DWI.^1^ A clinico-radiological diagnosis of lateral cavernous wall epidermoid with posterior fossa extension was made, for which surgical excision was the treatment of choice. Common approaches for these lesions include frontotemporal craniotomy and tumor excision by Dolenc’s approach. In this video, we demonstrate the recently popularized novel surgical approach for paramedian skull base lesions.^2–4^ The endoscopic-assisted transorbital approach for excision of paramedian epidermoid with posterior fossa extension.

### 2:27 Surgical Procedure.

Patient was positioned in a supine with head end rotated toward the surgeon and elevated 15°. In our case, we used an endoscopic holder to stabilize the image and freeing of both hands for surgical manipulation. The transorbital approach started with a superior eyelid crease incision following the natural line, extending laterally to the zygomatic bone. The incision was made through the orbicularis muscle and followed through the avascular plane. The periosteal incision was first made on the lateral orbital rim and followed through superiorly. The subperiosteal plane along the lateral orbital rim was carefully followed into the periorbita. A periosteal incision was made on the lateral orbital rim on infratemporal side. The prefixation of the lateral orbital rim was completed, and superior and inferior osteotomies along the lateral orbital rim were carried out. Posterior osteotomy was made and orbital rim was removed in toto. The further dissection of the periorbital region was carried out medially along the greater wing of the sphenoid. The orbitofacial and zygomatic vessels were frequently encountered during this dissection and carefully coagulated and cut. The periorbita was dissected posteriorly until reaching the superior orbital fissure and the inferior orbital fissure. The posterior part of the lateral orbital wall was meticulously drilled away using a 4 mm, both diamond and cutting burr. Removing the lateral orbital rim aids in better instrumentation and reduces pressure on the orbit. The greater wing of the sphenoid was drilled to access the dura of the middle cranial fossa, and the bony junction between the lateral wall and the roof of the orbit was also drilled to enhance visualization of the meningo-orbital band.

The sagittal crest,^5^ a triangular bone part of the sphenoid’s greater wing, is situated dorsal to the superior orbital fissure and reaches inferiorly to the foramen rotundum. It has been carefully dissected and drilled out. One must be cautious when using long-shaft rotating burrs, as they can easily catch the orbital and infratemporal fossa tissues, compromising the corridor. The meningo-orbital band was coagulated and cut, facilitating the dissection of the temporal dura from the cavernous sinus area. The pearly white tumor capsule was identified and dissected posteriorly and inferiorly following the dural plane. The sagittal crest’s inferior part is lowered to establish an inferior limit of the tumor.

### 5:46 Removal of Tumor.

After exposure to the anterolateral tumor capsule, it was incised, and the cavity was filled with soft, granular material that was often easily suckable. Using a fine periosteal elevator, the tumor content was carefully dissected from the floor, temporal lobe, and cavernous side. At the same time, the dissected tumor was suctioned out. After clearing the tumor from the superior aspect of the petrous bone, there was dehiscence over the horizontal petrous carotid. Using a ring curette, further dissection of the tumor from the posterior fossa, petrous bone, temporal lobe, and over the cavernous wall was carefully performed. The curved suction was used to remove a tumor from the posterior fossa, and the cerebellum and posterior circulation were visualized. After adequate tumor debulking, the cavity was well visualized, and the anterior venous bleed was controlled with gelatin sponges and supported with neuro patties. There were sticky tumor capsules along the cavernous sinus and temporal dura. Some were carefully dissected, while some parts of the capsules were left to avoid unwanted complications. The posterior fossa and tumor cavity were again visualized for hemostasis and irrigated with saline. The end cavity surface was covered with gelatin sponges and the fat that was covered with oxidized cellulose sheets. These were used to obliterate the anterior part of the tumor cavity and the posterior periorbital space. In the end, the resected prefixed lateral orbital rim was secured using screws. Skin and subcutaneous tissue were closed in layers using monofilament absorbable sutures.

### 8:11 Postoperative Course.

The postoperative period was uneventful, and the patient was discharged on postoperative day 3 with no new-onset neurological deficit or wound complication. The 3-month follow-up showed excellent cosmetic, functional, and neurological outcomes, with complete excision of the tumor. On histopathology, the tumor was confirmed as an epidermoid. On 3-month follow-up MRI, cerebrospinal fluid filled the cavity with no gross residual disease noted and the patient resumed her daily routine activities.

Thank you.

## Disclosures

The authors report no conflict of interest concerning the materials or methods used in this study or the findings specified in this publication.

## Author Contributions

Primary surgeon: AK Jaiswal, Manogaran. Assistant surgeon: Shukla, Chithambaram. Editing and drafting the video and abstract: AK Jaiswal, Manogaran, Shukla, Chithambaram, Mehrotra, Das K. Critically revising the work: AK Jaiswal, Manogaran, Chithambaram, Mehrotra, S Jaiswal. Reviewed submitted version of the work: AK Jaiswal, Manogaran, Mehrotra, Das K, S Jaiswal. Approved the final version of the work on behalf of all authors: AK Jaiswal. Supervision: AK Jaiswal, Manogaran, S Jaiswal.

## Supplemental Information

### Patient Informed Consent

The necessary patient informed consent was obtained in this study.
